# Efficacy of Small and Repeated Doses of Calomel

**Published:** 1848-11

**Authors:** 


*Efficacy of Small and Repeated Doses of Calomel.* By Prof. TROSSEAU. —Prof. T. has been much in the habit, for some time past, of administering in *acute* diseases, as rheumatic fever, puerperal peritonitis, iritis, &c., minute doses of calomel, and with complete success, He takes one grain of c. and one drachm of sugar, and divides it into twenty-four powders, one of which is taken every hour. The same treatment is continued two, three, or more days, or until the gums are touched. This usually occurs in 48 hours, and sometimes even in 24 hours, or after the administration of only one grain of calomel. It is rarely necessary to continue the powders during the third day.

The advantages of this method are—1. The system is brought under the influence of mercury as rapidly and as certainly as by any other mode of administering the drug. 2. The mercurial action never proceeds further than is intended, and the serious consequences of excessive salivation are entirely avoided.

In the treatment of puerperal peritonitis, M. Velpeau formerly used large doses of calomel and rubbed in mercurial ointment very freely, but unpleasant effects often followed this practice. But of late Prof. T. has adopted the plan of small and repeated doses, and he is convinced that he thus obtains all the good, and completely avoids the evil effects of the remedy.

In *chronic* diseases the same doses may be given at longer intervals; tenderness of the gums will not probably appear before the fifth or eighth day.

This method of giving calomel originated with Dr. Law of Dublin, who proposed it a few years since, but it did not at the time appear to receive much notice. The *modus operandi* of this method appears, according to Mialhé, to consist in the fact that calomel in the stomach undergoes a slow change before absorption, by coming in contact with an alkaline solution, as that of common salt, which thus partially changes it into a bi-chloride and metallic mercury. Hence as only small portions of calomel can be thus converted, it is immaterial, so far as absorption is concerned, whether one grain or one drachm is administered, as in either case the quantity of bi-chloride formed is the same. According to this view, the exhibition of common salt with the calomel ought to increase its activity in a marked degree, and perhaps the cases of so-called idiosyncrasy, where a small quantity of chloride has given rise to severe salivation, may find in this circumstance a rational explanation.— *Wood's Retrospect.*

